# Distribution of ETBE-degrading microorganisms and functional capability in groundwater, and implications for characterising aquifer ETBE biodegradation potential

**DOI:** 10.1007/s11356-021-15606-7

**Published:** 2021-08-04

**Authors:** Henry C. G. Nicholls, Stephen A. Rolfe, Helen E. H. Mallinson, Markus Hjort, Michael J. Spence, Matthijs Bonte, Steven F. Thornton

**Affiliations:** 1 Groundwater Protection and Restoration Group, Department of Civil and Structural Engineering, University of Sheffield, S1 3JD, Sheffield, UK; 2 Department of Animal and Plant Sciences, Alfred Denny Building, University of Sheffield, S10 2TN, Sheffield, UK; 3 Concawe, Boulevard du Souverain 165, 1160 Brussels, Belgium; 4 Shell Global Solutions International B.V., Rijswijk, 2288GK, The Netherlands; 5 Present Address: British Geological Survey, Environmental Science Centre, Keyworth, Nottingham, NG12 5GG UK; 6 Present Address: Ministry of Infrastructure and Water Management, The Hague, The Netherlands

**Keywords:** Ethyl *tert*-butyl ether, Bioremediation, Aquifer microorganisms, Attached community, *ethB* gene

## Abstract

Microbes in aquifers are present suspended in groundwater or attached to the aquifer sediment. Groundwater is often sampled at gasoline ether oxygenate (GEO)-impacted sites to assess the potential biodegradation of organic constituents. However, the distribution of GEO-degrading microorganisms between the groundwater and aquifer sediment must be understood to interpret this potential. In this study, the distribution of ethyl *tert*-butyl ether (ETBE)-degrading organisms and ETBE biodegradation potential was investigated in laboratory microcosm studies and mixed groundwater-aquifer sediment samples obtained from pumped monitoring wells at ETBE-impacted sites. ETBE biodegradation potential (as determined by quantification of the *ethB* gene) was detected predominantly in the attached microbial communities and was below detection limit in the groundwater communities. The copy number of *ethB* genes varied with borehole purge volume at the field sites. Members of the Comamonadaceae and Gammaproteobacteria families were identified as responders for ETBE biodegradation. However, the detection of the *ethB* gene is a more appropriate function-based indicator of ETBE biodegradation potential than taxonomic analysis of the microbial community. The study shows that a mixed groundwater-aquifer sediment (slurry) sample collected from monitoring wells after minimal purging can be used to assess the aquifer ETBE biodegradation potential at ETBE-release sites using this function-based concept.

**Supplementary Information:**

The online version contains supplementary material available at 10.1007/s11356-021-15606-7.

## Introduction

Ethyl *tert*-butyl ether (ETBE) is a gasoline additive that belongs to a broader group of chemicals known as gasoline ether oxygenates (GEOs). GEOs are used in fuels to increase the octane rating, enhance fuel combustion and reduce emissions. Methyl *tert*-butyl ether (MTBE) is the most commonly used GEO worldwide, although ETBE is used increasingly in European markets, supporting the requirements of the EU Renewable Energy Directives (2009/28/EC) and greater use of biofuels. ETBE synthesised from (bio)ethanol meets this criteria and is added to gasoline formulations at up to 15 vol% (Schuster et al. 2012).

Accidental releases of ETBE into the subsurface environment, either as a pure chemical, or in a mixture (as found in gasoline formulations), can result in contamination of groundwater (Stupp et al. 2012; van der Waals et al. 2018). The potential for ETBE biodegradation in groundwater is determined largely by the presence and activity of organisms within the aquifer microbial community able to mineralise the parent compound. There are a limited number of organisms known to biodegrade ETBE (reviewed in Thornton et al. 2020) to completion, such as *Aquincola tertiaricarbonis* L108 (Rohwerder et al. 2006), or partially to *tert*-butyl alcohol (TBA), the intermediate metabolite of aerobic ETBE biodegradation, such as *Rhodococcus* sp. IFP 2042 (Le Digabel et al. 2013). The *ethABCD* gene cluster was identified as involved in ETBE biodegradation (Chauvaux et al. 2001). This gene cluster includes a cytochrome monooxygenase (*ethB*) that initiates the incorporation of molecular oxygen into ETBE. Transcriptional investigations revealed that this gene is upregulated in the presence of ETBE (Malandain et al. 2010). Therefore, the detection of the *ethB* gene in aquifer microorganisms demonstrates the presence of aerobic ETBE biodegradation potential in groundwater at an ETBE-release site (Fayolle-Guichard et al. 2012; Kucharzyka et al. 2019; Kyselková et al. 2019). While ETBE biodegradation facilitated by *ethB* is the best characterised route for aerobic biodegradation, other biodegradation pathways have been proposed, although the genes involved have not been identified (Rosell et al. 2012; Le Digabel et al. 2013; Gunasekaran et al. 2014).

The sampling of groundwater for both hydrochemical and microbiological analysis usually involves the collection of filtered, sediment-free water samples (Environment Agency 2003a, b; Imfeld et al. 2011; Hose and Lategan 2012; O’Dwyer et al. 2014; Korbel et al. 2017; USEPA 2017; Environmental Protection Authority 2019). The chemistry and microbiological community profile of groundwater in monitoring wells often differs significantly from groundwater in the adjacent aquifer (Kozuskanich et al. 2011; Sorensen et al. 2013; Roudnew et al. 2014). For these reasons, sampling protocols typically recommend pumping (purging) monitoring wells to reduce sample turbidity and draw fresh groundwater from the aquifer for sampling (Nielsen and Nielsen 2006; Harter et al. 2014; USEPA 2017). This approach aims to exclude the collection of aquifer sediment and obtain a groundwater sample that is considered representative of in situ conditions (Warren 2005; Cullimore 2007; Korbel et al. 2017). However, a significant proportion of microorganisms in the subsurface environment are attached to sediment/mineral surfaces (Alfreider et al. 1997; Williamson et al. 2012; Gregory et al. 2014; Ugolini et al. 2014a; Thornton et al. 2016). Planktonic (suspended) bacteria in groundwater are typically present in much lower numbers than attached microorganisms, for example 10^2^-10^6^ cells mL^-1^ and 10^4^-10^9^ cells g^-1^, respectively (as reviewed by Smith et al. (2018)). There may also be significant differences in the physiology, composition, structure and activity between suspended and attached communities (Lehman et al. 2001, 2004; Rizoulis et al. 2013; Anantharaman et al. 2016; Thornton et al. 2016; Smith et al. 2018). Sampling only the groundwater can therefore under-represent, or even exclude, important information concerning the attached microbial community in aquifer biofilms (Alfreider et al. 1997; Rizoulis et al. 2013; Ugolini et al. 2014a; Smith et al. 2018). Furthermore, the relative abundance of bacteria and composition of the suspended microbial community can vary temporally in pumped groundwater samples (Kozuskanich et al. 2011; Sorensen et al. 2013; Roudnew et al. 2014). Given that the proportion of groundwater and aquifer sediment changes during purging of monitoring wells, bias may therefore occur in the sampling of organisms with different distributions between these components (Lehman 2007). This can lead to differences in measurements of microbial activity and functional capability, with incorrect interpretation of important functional processes within the aquifer microbial community (Alfreider et al. 1997; Lehman et al. 2004; Handley et al. 2012; Rizoulis et al. 2013; Korbel et al. 2017; Smith et al. 2018). These issues can limit the reliability of microbiological investigations where the origin of organisms in groundwater or the characterisation of aquifer organisms and microbial communities involved in contaminant biodegradation is of interest (Cullimore 2007; Lebron et al. 2011; Rizoulis et al. 2013; Somaratne and Hallas 2015; Thornton et al. 2016; Korbel et al. 2017).

To our knowledge, no studies have examined the physical location of ETBE-biodegrading activity in an aquifer, that is, the relative contribution of suspended and attached microbial communities to this potential. However, is it known that the taxonomy of attached and suspended microbial communities in aquifers can differ (Alfreider et al. 1997; Rizoulis et al. 2013; Hug et al. 2015; Smith et al. 2018; Fillinger et al. 2019). Interestingly, several studies have investigated the colonisation of functionally-important GEO-degrading organisms on inert surfaces used in bioreactors (Kharoune 2001; Purswani et al. 2011; Hicks et al. 2014; Alfonso-Gordillo et al. 2016; Guisado et al. 2016). These organisms were isolated from GEO-release sites and it is a reasonable hypothesis that they will preferentially attach to surfaces in aquifers. Sampling groundwater may therefore give an incomplete view of the overall potential for ETBE biodegradation due to these biases. The extent to which such biases are a problem is unknown. However, a strong preference for attachment would lead to a systematic under-sampling of these organisms in groundwater and inaccurate assessments of biodegradation potential.

The aim of this study was to determine the location of ETBE-degrading organisms and functional capability for ETBE biodegradation within groundwater and aquifer sediment fractions, to support the interpretation of ETBE biodegradation potential at ETBE-impacted sites. This is necessary where a microbiological assessment may be used in addition to the groundwater hydrochemical assessment that is normally undertaken at a gasoline-impacted site (ASTM 1998; Wiedemeier et al. 1999; Environment Agency 2000; API 2007). This is also important given that different purging regimes may be implemented in the sampling process (Nielsen and Nielsen 2006; CL:AIRE 2008; Harter et al. 2014; USEPA 1996, 2017) and that for some aquifer settings (e.g. bedrock aquifers) it may only be possible to collect groundwater samples for microbiological characterisation (Itävaara et al. 2011; O’Dwyer et al. 2014; Ben Maama et al. 2015; Eriksson et al. 2016; Wu et al. 2016).

To achieve this, laboratory studies of ETBE biodegradation and aquifer samples collected from two ETBE-release field sites were investigated using quantification of the *ethB* gene and culture-independent molecular analysis. Aerobic biodegradation of ETBE in the groundwater (planktonic) and aquifer sediment (attached) microbial communities was assessed in laboratory microcosm studies, and field samples from pumped monitoring wells at the ETBE-release sites were collected for comparison. All laboratory and field samples were processed to separate the planktonic and attached microbial communities, the location of *ethB* gene-containing organisms identified, and the microbial community composition analysed by high-throughput 16S rRNA gene sequencing. Pumped groundwater samples were also collected from monitoring wells at the field sites after purging 1, 3 and 6 well volumes to assess the effect of purging protocols on the detection of ETBE-degrading organisms. To the best of our knowledge, this is the first study conducted using laboratory experiments, supported by field samples, to determine the location of ETBE-degrading organisms in ETBE-impacted aquifers.

## Materials and methods

**Fig. 1:**
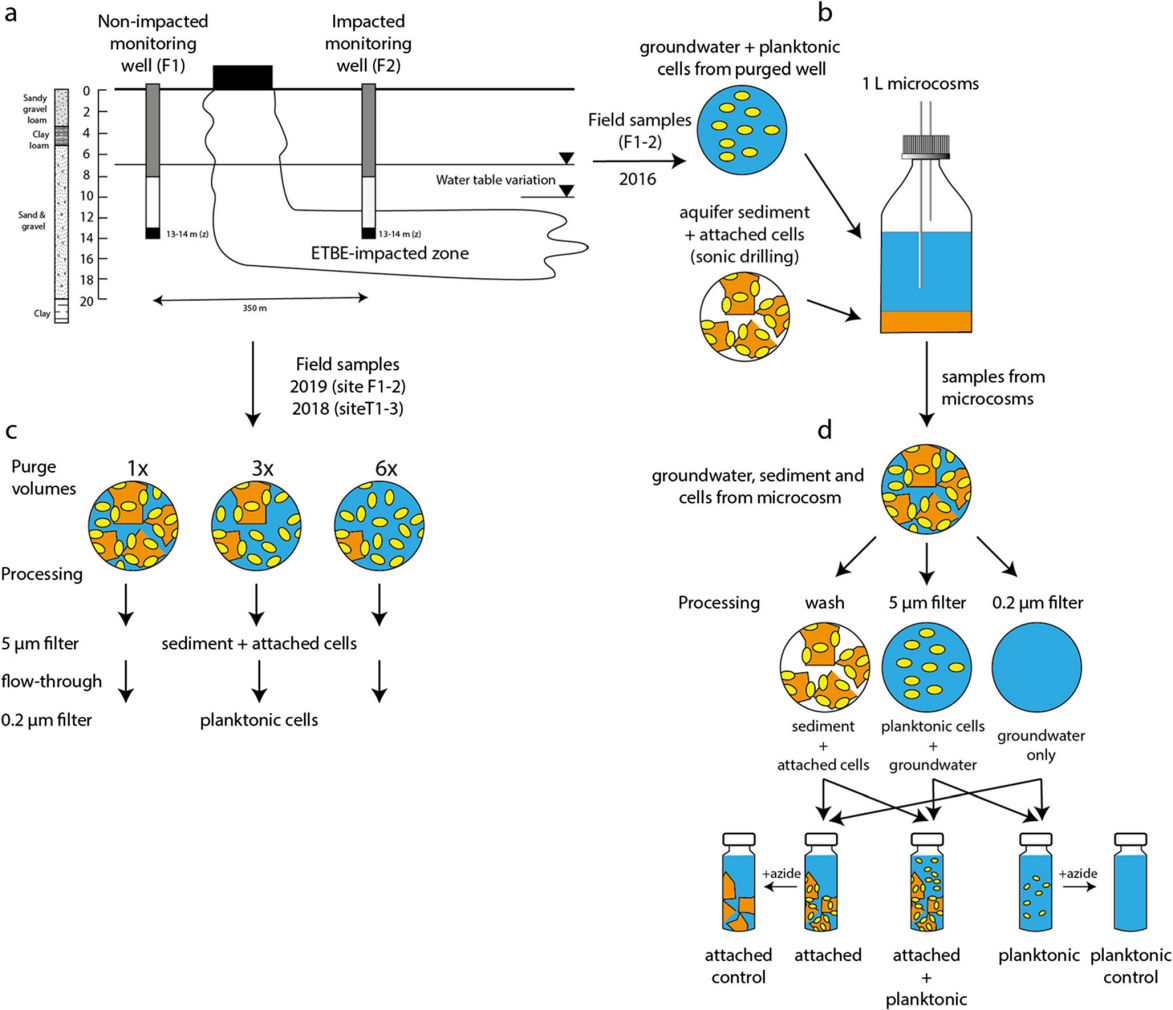
**A**) Diagram of Site F with monitoring wells installed at locations impacted (F2) and not impacted (F1) by ETBE contamination. Samples F1 and F2 were taken from 13 to 14 m below ground level. **B**) Microcosms containing attached and planktonic cells were assembled using groundwater (from wells F1 and F2) and aquifer sediment sampled from cores (collected by sonic drilling) at a 4:1 ratio. **C**) Aquifer sediment and groundwater mixtures obtained from monitoring wells at Site F (F1 and F2) and Site T (T1, T2 and T3, not shown) from different purge volumes. Aquifer sediment + attached cells was collected on 5 μm filters. Planktonic cells in the flow through were collected on 0.2 μm filters. **D**) Samples from B) were used to provide mixed groundwater + aquifer sediment. Groundwater without planktonic cells was obtained by filtration through a 0.2-μm filter. Groundwater + planktonic cells was obtained by filtration through a 5 μm filter. Sediment + attached cells was obtained by gently washing the sediment in 0.2 μm-filtered groundwater. These samples were used to establish 50 mL microcosms containing groundwater with planktonic cells only, attached cells only, or both attached + planktonic cells. Abiotic controls were created by the addition of sodium azide

The experimental programme developed for this research is summarised in Fig. 1, which shows the laboratory and field studies at the different study sites, the respective sampling schedule and materials collected. The procedures used to process these samples are explained below.

### Field site geology, hydrogeology and sample collection

Groundwater and aquifer sediment samples used to construct the microcosm experiments were collected from an ETBE-impacted site in France (Site F), as described in Nicholls et al. (2020). The aquifer comprised Quaternary alluvial deposits with up to 3 m sandy gravel loam underlain by gravel and sand, with a water table that fluctuates between 7 and 10 m below ground level. Two locations were selected to provide inocula for the laboratory experiments. These comprised a non-impacted location (F1), upgradient of the ETBE-impacted zone where ETBE and BTEX compounds were below detection limits, and an impacted location (F2) in the ETBE-impacted zone. Cored samples of aquifer sediment were collected by sonic drilling from these locations (350 m apart). Groundwater samples were collected from monitoring wells installed in the aquifer at locations F1 and F2 after coring. These samples were collected in autoclaved glass bottles, filled completely and stored at 4 °C until used to establish microcosms (Fig. 1).

To evaluate the effect of well purging, slurry (mixed groundwater-aquifer sediment) samples were obtained from two field sites. At Site F in France a slurry sample was collected from monitoring wells F1 and F2 after 1, 3 and 6 purge volumes, using a submersible pump at a flow rate of 6 L min^-1^. The slurry samples were collected in autoclaved 500 mL glass bottles, filled completely and stored at 4 °C. Neither well F1 nor F2 had detectable ETBE at the time of sampling (February 2019). The groundwater fraction from wells F1 and F2 was sampled for the analysis of inorganic determinands (Table S1).

Similar slurry samples were also collected from a second ETBE-impacted site in Turkey (Site T). At this site the aquifer comprises a 1 m surface clay layer underlain by 3-4 m sand and silt. The monitoring wells are installed at a maximum depth of 3-4 m, and the water table fluctuates between 1 m and 2.5 m below ground level. Slurry samples were obtained from pumped monitoring wells (T1-3) using a submersible pump at a flow rate of 0.3 L min^-1^, following the same purging regime and storage procedure used at Site F. The groundwater from all three wells contained ETBE at the time of sampling (November 2018): 7.4, 0.18 and 0.14 mg L^-1^ for T1, T2 and T3, respectively. The groundwater fraction from wells T1-3 was also sampled for the analysis of inorganic determinands (Table S1).

### Planktonic and attached microbial community microcosm experiments

Large volume (1 L) laboratory microcosms were constructed with groundwater and aquifer sediment sampled at Site F (Fig. 1b), as described in Nicholls et al. (2020). A set of 50 mL microcosms, in which the planktonic and attached microbial communities were separated as in Fig. 1d, was then created from the 1 L microcosms containing inoculum sampled between 13-14 meters below ground level, using the following components:

1. ETBE-contaminated groundwater without microbial cells or sediment fines, obtained by filtration through a 0.2 μm Whatman polycarbonate filter;
2. ETBE-contaminated groundwater with planktonic microbial cells but without sediment fines, obtained by filtration through a 5-μm Whatman polycarbonate filter;
3. Aquifer sediment with attached microbial cells, prepared by gently washing sediment with 2 volumes of 0.2 μm-filtered ETBE-contaminated water.

“Planktonic” microcosms were prepared by mixing 20 mL of groundwater + planktonic cells (#2) with an equal volume of groundwater without planktonic cells (#1). “Attached” microcosms were prepared using 20 mL of washed aquifer sediment + attached cells (#3) with an equal volume of groundwater without planktonic cells (#1). Triplicate planktonic and attached microcosms were prepared in autoclaved 50 mL glass vials sealed with aluminium crimp-caps, with sterile controls created by adding 2 g L^-1^ (w/v) sodium azide (Shah et al. 2009). An additional biotic control (“Attached + Planktonic”) to compare ETBE biodegradation in a mixed groundwater-sediment microcosm was created by mixing 20 mL of washed aquifer sediment + attached cells (#3) with 20 mL of 5 μm-filtered ETBE-contaminated groundwater + planktonic cells (#2). ETBE (99 % purity, Sigma) was added at a nominal concentration of 1.2–1.8 mg L^-1^. The microcosms were incubated in the dark at 12 °C to reflect the mean groundwater temperature of European sites (Tissen et al. 2019). Given the duration of the experiment (<30 days), no significant fluctuation in the sample site groundwater temperature is expected over this timeframe.

Groundwater samples were taken at intervals for the analysis of ETBE. Prior to sampling all microcosms were removed from the incubator, mixed gently and placed at room temperature for one hour. A 1-mL groundwater sample was extracted using a sterile syringe and needle and analysed immediately using GC-MS (“Geochemical analysis**”** section). Once the experiment was complete planktonic microbial cells were harvested by filtering the groundwater through a 0.2 μm filter (PES, Millex), which was then used for DNA extraction (“Molecular analysis of microcosms**”** section). DNA from attached cells was extracted directly from the aquifer sediment samples.

### Field samples

Slurry samples obtained after the removal of different purge volumes in pumped monitoring wells at Site F and Site T were processed in a similar manner to samples from the microcosm experiments. Sample bottles were inverted to re-suspend all the sediment immediately prior to sampling. The slurry was filtered through a 5-μm filter to capture the aquifer sediment and attached microbial cells (Fig. 1c). The groundwater and planktonic cells passing through this filter were collected in a sterile 50 mL Falcon tube and the planktonic cells then harvested from this liquid on a 0.2 μm filter. Membranes were stored at -80 °C prior to DNA extraction.

### Geochemical analysis

The chemical composition of groundwater samples was analysed using methods described in Nicholls et al. (2020). The analysis of ETBE and TBA was performed by solid phase micro-extraction (SPME) of the aqueous phase using a CombiPAL autosampler (CTC Analytics AG, Zwingen, Switzerland) connected to a Shimadzu QP1000 GC-MS. A 85 μm Carboxen/PDMS StableFlex SPME fibre (Supelco, UK) was used for sample extraction, with an extraction time of 2 min. The fibre was desorbed in the injection port of the GC-MS at 300 °C for 3 min. The GC-MS was fitted with a 20 m DB-624 column (121-1324, Agilent Technologies Ltd), with an initial oven temperature of 40 °C. The temperature programme was increased at 10 °C min^-1^ to 170 °C, then at 40 °C min^-1^ to 250 °C, then held for 2 min, for a total run time of 17 min. The column flow was 1.18 ml min^-1^, using Helium as the carrier gas, with a split ratio of 30:1. The GC-MS interface was set to 250 °C, with the GC-MS ion source at 200 °C and solvent cut time of 1.4 minutes. The MS programme was set to Scan/SIM mode, allowing for a full scan of the m/z values 30-200 together with monitoring of selected ions corresponding to the retention times of each analyte. In addition to calibration standards, an internal standard containing deuterated isotopologues of the analytes of interest was also prepared. The internal standards were added to all samples prior to analysis. The peak area represented by the quantification ion was used to calculate the concentration of the analyte in the original sample. The ratio of the peak areas of the quantification and reference ions was used together with the retention time to identify the analyte peak, using GC-MSsolution V2 software (Shimadzu). Dissolved major ions were analysed using a Dionex 3000 instrument equipped with cation and anion modules for simultaneous detection, as described in Nicholls et al. (2020).

### Molecular analysis of microcosms

DNA was extracted using a FastDNA Spin kit for Soil (MP Biomedicals, UK) according to the manufacturer’s instructions, with an additional 10 min incubation at 65 °C prior to homogenisation. DNA quantification was performed using a Qubit dsDNA HS Assay (ThermoFisher, UK).

#### Quantitative real-time PCR (qRT-PCR)

Absolute quantification of *ethB* and 16S rRNA genes was carried out using standards and qRT-PCR, as described in Nicholls et al. (2020).

#### 16S rRNA gene sequencing

16S rRNA genes were amplified, purified and sequenced according to Nicholls et al. (2020). Each sample was amplified in triplicate to minimise PCR bias and pooled prior to sequencing using Illumina MiSeq. DNA sequences were supplied as demultiplexed FASTQ data files containing 250 bp paired end sequences. Initial data processing was performed using Qiime2 (Bolyen et al. 2019) to produce a biom file of Operational Taxonomic Units (OTUs), abundances and taxonomy. Raw sequences were processed using dada2 to remove primers, chimeric sequences and produced paired sequences (Callahan et al. 2016). Samples contained between 12,209 and 254,448 reads after processing.

Sequences were aligned using MAFFT (Katoh et al. 2019) and a rooted phylogenetic tree produced using FastTree2 (Price et al. 2010). Sequences were classified using Scikit-learn (Pedregosa et al. 2011) trained against the V3-V4 regions of 16S rRNA genes in the SILVA132 database (Quast et al. 2012). Further analysis was performed in R (R Core Team 2019). The Qiime2 biom artefact produced above was loaded into R using functions from the ‘qiime2R’ package and processed using the ‘phyloseq’ package (McMurdie and Holmes 2013). For analyses requiring even sampling depth, samples were normalised to 10,000 reads per sample.

### Microscopy

Total cell counts of planktonic bacteria were performed using 100 μL of groundwater sample, mixed with 10 mL of 10 mM NaCl and vacuum-filtered through a black 25.4 mm diameter, 0.2 μm filter membrane (Whatman). Bacteria were stained using 200 μL of 6 μM SYTO 9 Green fluorescent dye (Thermo) and incubated in the dark for 15 min. The cell counts were performed using a Leica DM6 fluorescence microscope. Cells were counted under a 40× objective and the mean counts in 5 randomly selected field of views are presented. Nicholls et al. (2020) showed previously that the proportion of live planktonic cells from the same sample site ranged between 48 and 82 %.

Images of aquifer sediment grains were acquired from 5 μm- and 0.2 μm-filtered slurry samples, respectively. Sediment grains were gently scraped from each membrane surface using a sterile scalpel and were gently mixed with 20 μL ultrapure water on a microscope slide. Bright field z-stack images were obtained using a Leica DM6 fluorescence microscope.

### Statistical analysis

Statistical analysis was performed in R (version 4.03) (R Core Team 2020) using the linear model function ‘lm’ with interactions between location, pump volumes and filters. Only single samples could be obtained, hence comparisons were made between sites using the different wells (F1-F2, T1-T3) and filters (0.2 μm and 5 μm) as replicates. Molecular analyses were performed in triplicate, with the mean value of these technical replicates used for statistical analyses. Post-hoc comparisons were made using a Tukey test using the ‘emmeans’ package (Lenth 2021). All measurements were log transformed prior to analysis. If values were below the detection limit, then the detection limit was substituted before log transformation.

## Results

### ETBE biodegradation potential in planktonic and attached microbial communities from microcosm experiments

In the microcosm study described in Nicholls et al. (2020) groundwater and aquifer sediment was collected from two locations (F1—non-impacted and F2—ETBE-impacted) at Site F to examine the aerobic biodegradation of ETBE. At the time of sampling (October 2016) ETBE was detected only in well F2 (1.4 mg L^-1^). These 1 L microcosms were incubated for 200 days with periodic re-addition of ETBE. The F2 microcosms from this experiment were then used to create 50 mL microcosms to assess the contribution of attached and planktonic cells in ETBE biodegradation (see Fig. 1).

The aquifer ‘sediment + attached cells’ fraction was prepared by gently washing the sediment with 0.2 μm filter-sterilised groundwater. DNA yields from the 1 L F1 and F2 microcosms were 13.4 and 51 ng mL^-1^, respectively. The ‘planktonic cells’ fraction was collected by filtration through a 5 μm filter and capture on a 0.2 μm filter. DNA yields were much lower (0.01 ng mL^-1^ in both cases). These observations implied that most microbial cells were attached to the sediment fraction in the microcosms. From this, we formulated the alternative hypotheses that (a) the majority of ETBE-degraders would therefore be attached, but with no differences in their relative abundance, or (b) that in addition to the numerical differences in cell numbers, the relative abundance of ETBE-degraders might also be altered.

**Fig. 2:**
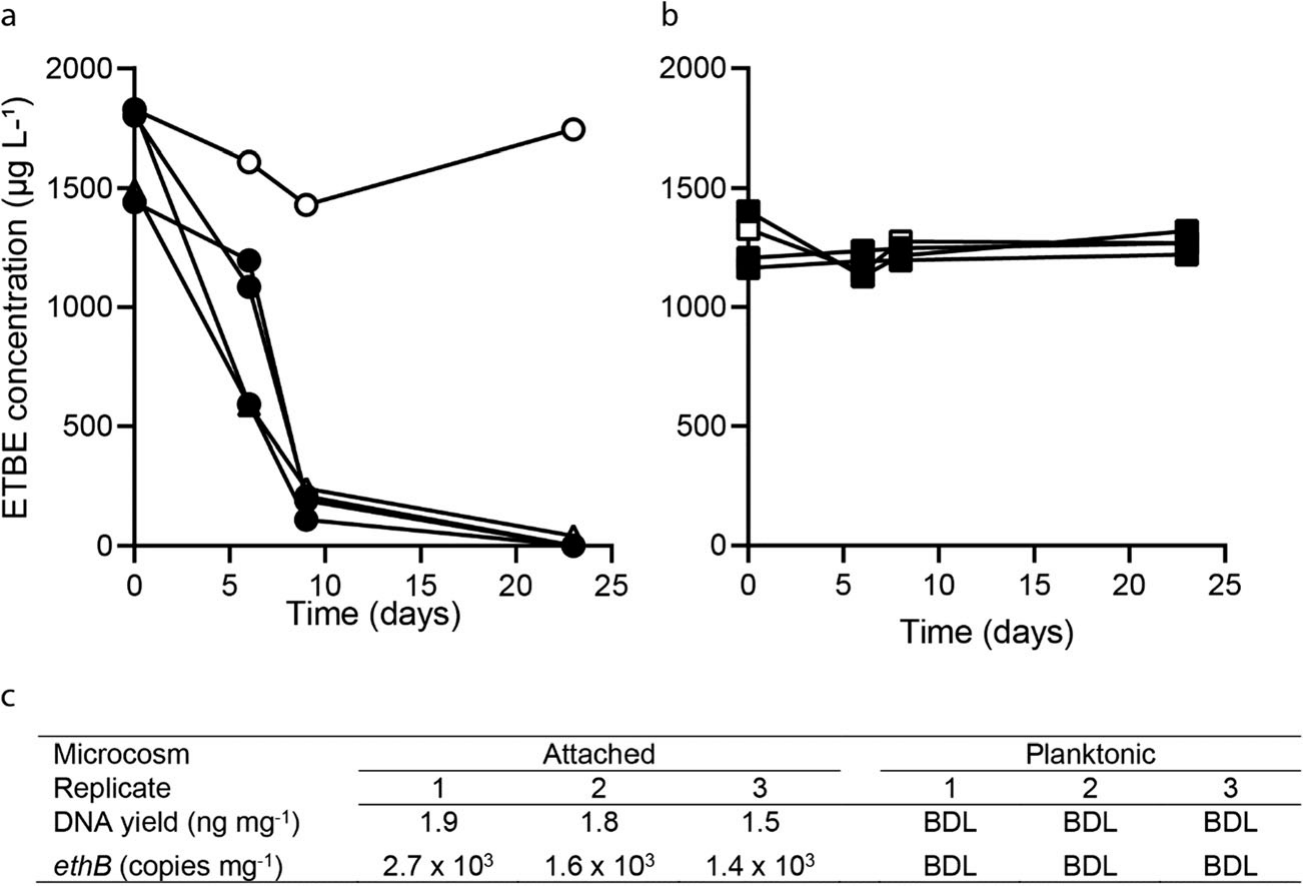
ETBE biodegradation in microcosms containing **a**) attached cells (circles) or attached + planktonic cells (triangles), or **b**) planktonic cells. Individual replicates are connected by lines. Live samples are shown as black symbols, whereas abiotic samples created by the addition of sodium azide are shown as white symbols. **c**) DNA yields and *ethB* gene copy numbers from replicate attached and planktonic microbial communities. BDL indicates below detection limit

ETBE was completely biodegraded after 23 days without a lag in the 50 mL ‘Attached’ microcosms containing the aquifer sediment and 0.2 μm filter-sterilised ETBE-contaminated groundwater (Fig. 2a). ETBE biodegradation in the ‘Attached’ microcosms and ‘Attached + Planktonic’ microcosms (containing aquifer sediment and 5 μm-filtered ETBE-contaminated groundwater) was 90% completed within 10 days. No TBA was detected during ETBE biodegradation, presumably due to rapid consumption of this metabolite by the consortia (Nicholls et al. 2020). No ETBE biodegradation was observed in the ‘Planktonic’ microcosms (containing the planktonic cells and 0.2 μm filter-sterilised ETBE-contaminated groundwater) or the abiotic controls (Fig. 2a and b). There was no detectable DNA yield from the ‘Planktonic’ microcosms (below detection limit of 0.01 ng μL^-1^), but comparable DNA yields from each ‘Attached’ microcosm. Similar results were found when *ethB* gene copy numbers were quantified using qRT-PCR (Fig. 2c). Therefore, the ETBE-degrading activity and associated organisms in these microcosms were found entirely in the microbial community attached to the aquifer sediment.

### ETBE biodegradation potential in planktonic and attached microbial communities in pumped groundwater samples from field sites

Pumped groundwater samples, collected after purging of monitoring wells at Site F and Site T, were compared with the laboratory microcosm samples processed and filtered in the same way. The monitoring wells F1 and F2 at Site F which had been used for the 1 L and 50 mL microcosm experiments (see above) were re-sampled.

Microscopy confirmed that the 5 μm filter captured most sediment grains in these field samples, with only a few fine grains (<5 μm) in the 0.2 μm-filtered samples (Supplementary Figure 1). Cell counts also showed that the planktonic cells passed through the 5 μm filter, as there was no reduction of cell numbers in the planktonic phase after 5 μm filtration (data not shown).

**Fig. 3:**
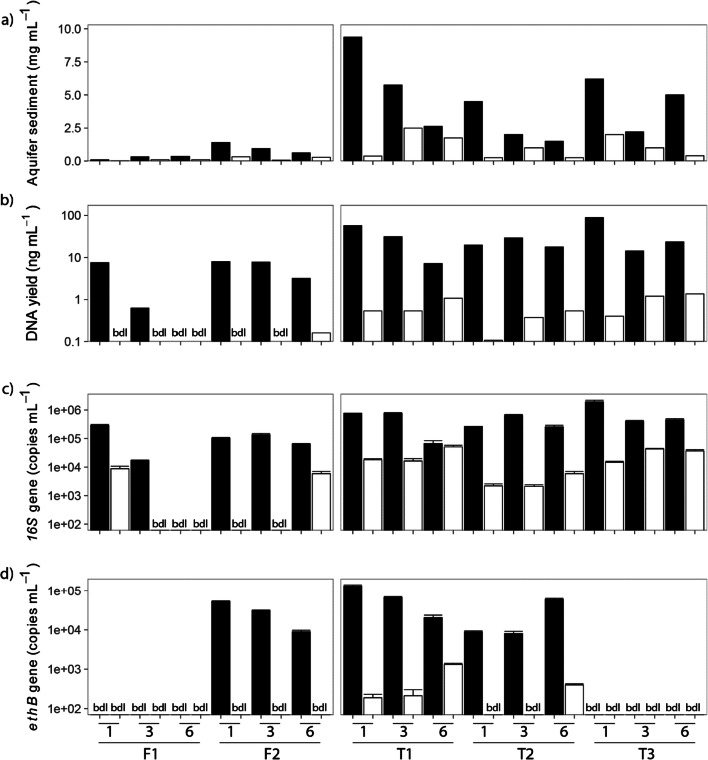
Analysis of aquifer sediment collected from an un-impacted groundwater monitoring well (F1) and an ETBE-impacted groundwater monitoring well (F2) at Site F and three ETBE-impacted monitoring wells (T1-3) at Site T, after purging 1, 3 or 6 borehole volumes of groundwater which was filtered through a 5 μm filter (black bars) followed by a 0.2-μm filter (white bars): (**a**) Concentration of aquifer sediment in bulk sample, (**b**) concentration of DNA extracted from filtered aquifer sediment, (**c**) 16S rRNA copy numbers, and (**d**) *ethB* gene copies determined by qRT-PCR. * bdl symbol indicates values were below detection limits. Where error bars are shown, results are the mean + SD of technical replicates

For all samples, more sediment was collected on the 5 μm filter than the 0.2 μm filter (*p* < 0.001) (Fig. 3). At Site F between 0.02 and 1.4 mg of sediment was collected per mL of sample. More sediment was collected from monitoring well F2 than F1, and the amount of sediment collected on the 5 μm filter decreased at monitoring well F2 as the number of purge volumes increased. The amount of fine sediment collected on the 0.2 μm filter was lower and unaffected by purge volume. At Site T more sediment was collected (0.25 to 9.4 mg mL^-1^) than at Site F (*p* < 0.001) and the amount of aquifer sediment collected on the 5 μm filter tended to decrease with increased purge volume, as expected from the purging process. While the aquifer sediment collected after purging 6 well volumes from monitoring well T3 was greater than that after purging 3 well volumes, this was still less than the first purge volume. This most likely reflects physical heterogeneity in the sediment size fractions sampled in the aquifer during pumping (Wu et al. 2013). However, in all wells at Site T, most aquifer sediment was collected after the first purge volume.

DNA could be extracted from all Site T samples and most of the 5 μm-filtered Site F samples analysed, but yields from 0.2 μm-filtered Site F samples were low or undetectable. DNA extracted from the 5 μm-filtered samples was derived solely from attached microbial cells, whereas DNA extracted from 0.2 μm-filtered samples was derived from both planktonic cells and microbes attached to fine (<5 μm) sediment particles, as confirmed by microscopy (Supplementary Figure 1). DNA yields were higher from the 5 μm-filtered samples, both in absolute terms (Fig. 3b) and DNA yield per mg sediment (~7 mg DNA mg^-1^ sediment in 5 μm-filtered samples compared to ~0.6 mg DNA mg^-1^ sediment in 0.2 μm-filtered samples) (*p* < 0.001). This indicates that a greater proportion of the microbial biomass was attached to larger sediment particles at both field sites. Generally, the greatest DNA yields were obtained from slurry samples collected after purging 1 or 3 well volumes, and less from samples collected after purging 6 well volumes, indicating that the early purge samples (up to 3 well volumes) are important for maximising DNA yields.

Total microbial numbers were estimated using the 16S rRNA gene copy number as a proxy. At Site T microbial cell numbers were higher in the attached than planktonic phase (*p* = 0.013), often by several orders of magnitude (Fig. 3b), confirming that most of the microbial community in the aquifer sample originates from the attached organisms. Little or no DNA was recovered from the 0.2 μm-filtered samples at Site F, confirming that most organisms were also attached to the aquifer sediment at this site. Quantification of the *ethB* gene, as a measure of ETBE-degrading organisms, was also carried out to determine the location of these organisms. The *ethB* gene was below detection limit in samples from the non-impacted well (F1) at Site F. It was detected only in 5 μm-filtered samples from well F2 at ~10^4^ gene copies mL^-1^ for all purge volumes, although detection of the *ethB* gene was highest in the first purge volume. At Site T, the *ethB* gene was detected in wells T1 and T2 only, at ~10^4^-10^5^ copies mL^-1^. The gene was detected in all 5 μm-filtered samples from wells T1 and T2, all 0.2 μm-filtered samples from well T1, but only after 6 purge volumes for the 0.2 μm-filtered sample in well T2. Where the *ethB* gene was detected in both the attached and planktonic phases, it was present at several orders of magnitude higher in the attached than the planktonic phase (*p* = 0.034).

**Fig. 4:**
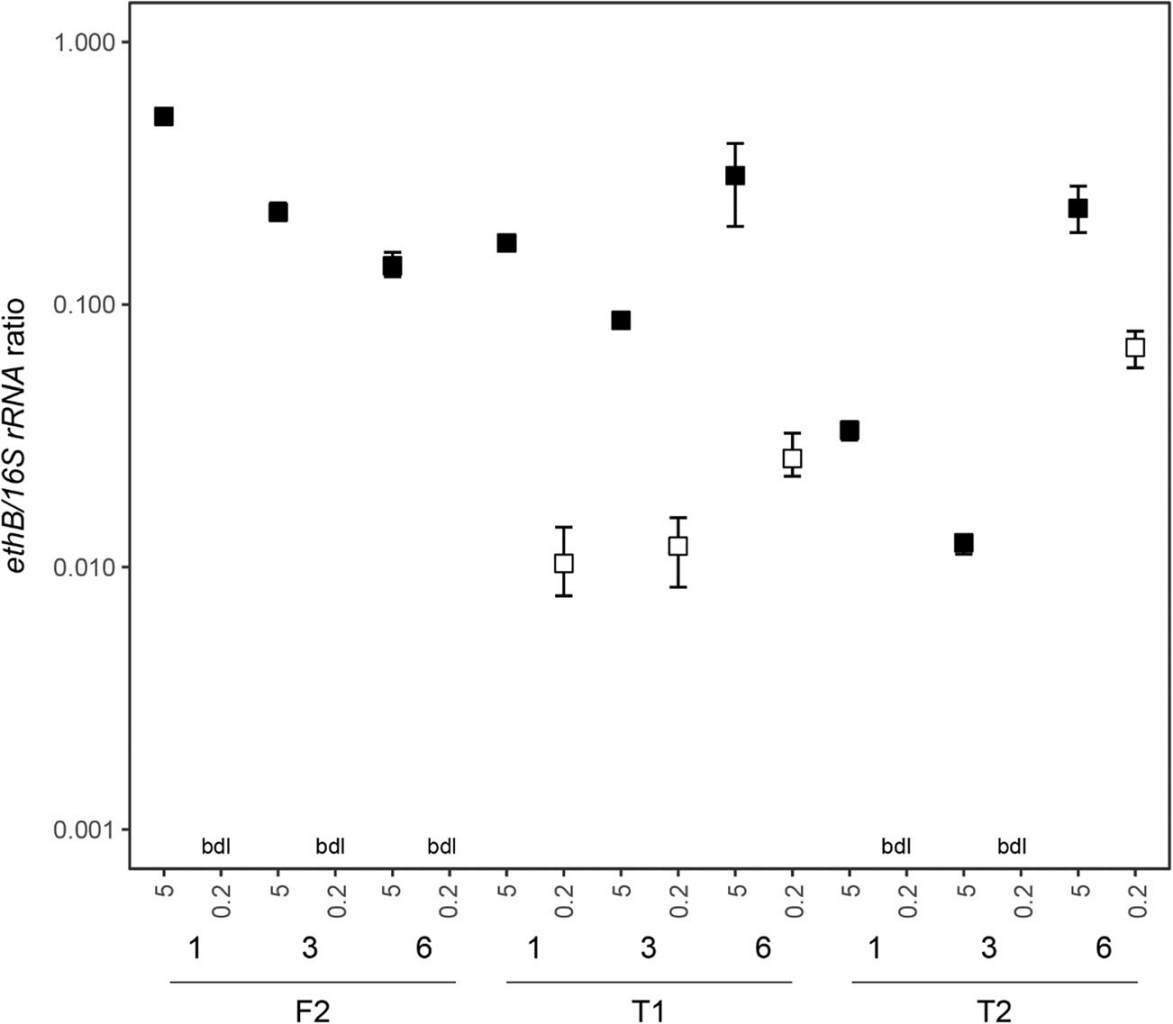
Plot of *ethB* to 16S rRNA gene copy number ratios for Site F, well F2, and Site T, wells T1 and T2, according to different purged borehole volumes (1, 3 or 6). Filled symbols represent the 5 μm-filtered samples and open symbols represent the 0.2 μm-filtered samples. * bdl indicates that the *ethB* gene was below detection limit and a ratio was therefore not determined. Results are the mean +/- SD of technical replicates

Subsequently, the *ethB*:16S rRNA gene copy number ratio was examined to deduce if the attached or planktonic fractions provided the most representative sample to determine ETBE biodegradation potential in these aquifers. At Site T the *ethB*:16S rRNA ratio was typically 10x higher in the 5 μm-filtered samples than in the 0.2 μm-filtered samples (*p* = 0.004). In wells F2 and T1, the ratio was > 0.1 for the 5 μm-filtered samples (Fig. 4). In all cases where the *ethB* gene was detectable in the 0.2 μm-filtered samples, it had an *ethB*:16S rRNA ratio < 0.1.

High-throughput 16S rRNA gene sequencing of the microbial communities from both field sites was performed. While community membership was similar between samples from the same monitoring wells, membership differed markedly (Supplementary Figure 2a). In general, ETBE-impacted monitoring wells from Site F (F2) and Site T (T1, T2, T3) were dominated by alpha-, delta- and gamma-Proteobacteria. The microbial community of the non-impacted monitoring well (F1) at Site F contained alpha and gamma-Proteobacteria but also Bacteroidia; the delta-Proteobacteria were much reduced (Supplementary Figure 3). Furthermore, while the microbial community membership of the attached and planktonic fractions was similar for each individual monitoring well (Supplementary Figure 2a), the relative abundance of these organisms changed with purging (Supplementary Figure 2b), confirming that the purging regime influenced the aquifer microbial community composition.

In the previous study (Nicholls et al. 2020) using microcosms constructed from Site F inocula, members of the Comamonadaceae, among others, were identified as organisms that increased in relative abundance (designated as ‘responders’) after exposure to ETBE. Regardless of the ETBE exposure history, members of the Comamonadaceae increased in abundance when exposed to ETBE. It was therefore hypothesised that this group of organisms was involved in ETBE biodegradation, either as primary degraders or as degraders of intermediate metabolites of ETBE. To determine if they were ubiquitous across the two ETBE-release sites sampled in this study, the OTUs identified in the microcosm study were compared with the field samples obtained from Site F and Site T (Supplementary Figure 4).

At Site F less than 1 % of the total microbial community in monitoring well F1 (non-impacted) were identified as ETBE-responding organisms, whereas >4 % of the total microbial community in monitoring well F2 (ETBE-impacted) were previously characterised ETBE-responding organisms. Borehole purge volume had little effect on the relative abundance of these organisms (Supplementary Figure 4). At Site T members of the Comamonadaceae were identified in all three monitoring wells, albeit at varying abundances. It was noted that the presence of the *ethB* gene was not correlated with identified ‘responders’, for example the greatest abundance of responders was identified in monitoring well T3, yet the *ethB* gene was below the detection limit. In all cases where ETBE-responding organisms were detected at >1 % relative abundance, most belong to the Gammaproteobacteria, in which members of the Comamonadaceae family are found. It should be noted that in the Nicholls et al. (2020) study, the Greengenes database was used for taxonomic identification, whereas in this study the Silva database was employed. Due to this change, the nomenclature of taxonomy differs between the two databases; Comamonadaceae are placed in the Betaproteobacteria class when using Greengenes, but are grouped in the Gammaproteobacteria class in the Silva database. While the nomenclature of taxonomy varies between the two studies, a 100 % sequence identity match was required.

## Discussion

### Groundwater monitoring to evaluate ETBE-biodegradation

Concentrations of ETBE, TBA and dissolved oxygen are typically monitored in groundwater at ETBE-release sites to demonstrate aerobic biodegradation of ETBE (Rosell et al. 2005; Fayolle-Guichard et al. 2012; Stupp et al. 2012; Bombach et al. 2015; van der Waals et al. 2018; Nicholls et al. 2020; Thornton et al. 2020). The detection of the *ethB* gene in aquifer samples can provide additional microbiological-based evidence to support the hydrochemical assessment, complementing other molecular tools which may be deployed to evaluate GEO biodegradation (e.g. Fayolle-Guichard et al. 2012; van der Waals et al. 2018; Kucharzyka et al. 2019; Kyselková et al. 2019). This is site specific and may be appropriate in cases where the hydrochemical assessment suggests that conditions are favourable for ETBE biodegradation but this is not observed, or the hydrochemical data is unclear, for example due to releases of multiple GEO. It should be noted that the presence of the *ethB* gene in an aquifer is evidence for ETBE biodegradation potential. However, the absence of this gene does not imply the absence of this potential, as ETBE biodegradation could occur via other uncharacterised routes (Kyselková et al. 2019).

This study has shown that ETBE biodegradation potential cannot be characterised in groundwater-only samples by analysis of the *ethB* gene. However, several field-based approaches are available to sample the attached microbial community in an aquifer (Lehman 2007). These include the following:

1. Recovery of cored aquifer material and direct sampling of attached microorganisms (Lehman 2007; Lehman et al. 2004; Kieft 2010; Wilkins et al. 2014; Kiaalhosseini et al. 2016);
2. Incubation of native aquifer material, surrogate geological media (e.g. quartz sand, crushed rock), inert substrates (e.g. granular activated carbon), artificial platforms (e.g. sampling coupons) or *in situ* microcosms within a borehole or monitoring well and sampling the attached communities established after colonisation (Alfreider et al. 1997; Mandelbaum et al. 1997; Griebler et al. 2002; Lehman et al. 2004; Reardon et al. 2004; Hendrickx et al. 2005; Kovacik et al. 2006; Lehman 2007; Kästner and Richnow 2010; Aslett et al. 2011; Handley et al. 2012; Rizoulis et al. 2013; Smith et al. 2018; Mujica-Alarcon et al. 2021); and
3. Mobilisation of attached microorganisms for sampling using downhole sonication techniques (Ugolini et al. 2014a; Close et al. 2020).

These methods typically require specialist equipment, are expensive, labour-intensive and time-consuming (Hendrickx et al. 2005; Lehman et al. 2004; Lehman 2007; Kieft 2010; Wilkins et al. 2014; Hug et al. 2015). The collection of core material is undoubtedly the gold standard for sampling the aquifer microbial community (Lehman 2007), but core recovery may be difficult in cohesionless sediments (e.g. sands and gravels) and is susceptible to fragmentation or contamination by the drilling methods used (Wilkins et al. 2014; Kiaalhosseini et al. 2016; Close et al. 2020), or requires elaborate measures to assess and avoid contamination during sampling (Lehman et al. 2004; Spence et al. 2005; Wilkins et al. 2014; Kallmeyer 2016; Friese et al. 2017). The destructive nature of core sampling also means temporal sampling is not possible for the same location (Lehman 2007; Wu et al. 2013). Methods using the incubation of materials within boreholes can provide similar results to core samples (Handley et al. 2012). However, results may also depend on the incubation time and compatibility between the material used with that in the aquifer, to ensure representative sampling (Lehman et al. 2004; Lehman 2007; Smith et al. 2018; Mujica-Alarcon et al. 2021). Hence, sampling methods which can potentially address these constraints are recommended (Smith et al. 2018). As shown in this study, the collection of a mixed groundwater and aquifer sediment slurry sample from a pumped monitoring well can provide reliable DNA yields from small sample volumes (e.g. 10-50 mL), which enable the detection of the *ethB* gene when present. Other studies have also proposed the collection of sediment fines in boreholes to sample attached microorganisms (Cardenas et al. 2008; Wu et al. 2013; Li et al. 2018). The current method offers advantages in enabling relatively rapid and repeat sampling of existing groundwater monitoring wells at a site. This can be implemented within the routine groundwater sampling of GEO-impacted aquifers to support the hydrochemical assessment undertaken. It may also be appropriate for sampling the aquifer microbial community when monitoring the efficacy of engineered remediation measures used for plume management (e.g. Wu et al. 2013). By simultaneously collecting both groundwater and aquifer sediment, it enables suspended and corresponding attached microorganisms to be sampled, as advocated in other studies (Griebler et al. 2002; Lehman 2007; Ugolini et al. 2014b; Smith et al. 2018). It may therefore provide a practical and cost-effective alternative to other borehole-based methods available to sample the aquifer microbial community. An important feature would be the availability of an established monitoring well network at a site for sampling. There is value in exploring the application of this sampling approach for other aquifer settings and contaminant-release scenarios, beyond that examined for ETBE in the current study. However, the method may not be appropriate in aquifers (e.g. crystalline rock sites) in which there is limited sediment input to the monitoring well during groundwater pumping.

### Location of ETBE-degrading organisms in aquifers

While it is generally accepted that the attached and suspended microbial communities differ markedly, there is less understanding of the functionality of each fraction (Herrmann et al. 2017), especially for GEO biodegradation. Typically, functionality has been inferred from the presence of specific functional genes in microbial consortia (Wu et al. 2013; Smith et al. 2018). In the present study *ethB* gene copy numbers were used as a direct indication of functional capability for ETBE biodegradation within the microbial community, and to infer the presence of ETBE-degrading organisms. Numerous studies have investigated GEO-degrading organisms that colonise a surface, for use in a bioreactor (Alfonso-Gordillo et al. 2016; Hicks et al. 2014; Kharoune 2001; Purswani et al. 2011), suggesting that these degraders can attach to inert surfaces. Fayolle-Guichard et al. (2012) investigated the use of a batch-fed pilot plant to treat ETBE-impacted groundwater and reported that *ethB* gene copy numbers decreased without continual feeding. In their experiment a mixed culture was fixed to perlite and added to a bioreactor, with amendments of ETBE and BTEX. While *ethB* gene copy numbers increased with ETBE additions, they rapidly decreased once the substrate was consumed. It should be noted that only the water was sampled in the time-course experiment, even though the ETBE-degraders were initially fixed to perlite. It is therefore plausible that most of the *ethB* gene-containing organisms remained attached, but the addition of ETBE increased the *ethB* gene-containing organisms in the groundwater. This increase was probably caused by the translocation of new ETBE-degraders via dispersal (i.e. new cells that were yet to colonise a surface) and therefore the ETBE-degraders were transiently detected at higher concentrations in the water, but the number of these genes decreased rapidly as the microbes began to attach to a surface. Along with ETBE-degraders, TBA-degrading organisms have also been identified as surface attachers (Aslett et al. 2011). While such studies have shown that GEO-degrading organisms can attach, the current study provides evidence that ETBE-degraders preferentially attach to surfaces.

Based on the results of the microcosm experiments and ETBE-release sites investigated in this study, we conclude that: a) most cells are attached, as the vast majority of extracted DNA was obtained from the aquifer sediment fraction, and b) the *ethB* gene was detected at higher gene copy numbers and the ethB:16S RNA ratio was higher in the aquifer sediment fraction, compared with the corresponding groundwater samples, i.e. the relative abundance of ETBE-degraders is greater in attached communities compared to planktonic communities. Therefore, sampling of the attached community is essential to determine the diversity and abundance of ETBE-degraders in the microbial community. Furthermore, given that the *ethB* gene was not detected in the planktonic fraction, but readily detected in the aquifer sediment fraction at Site F, it is concluded that a sediment-free groundwater sample will not provide a representative sample for the presence of the gene at the site. At Site T the *ethB* gene was detected in both the planktonic and attached phases, although this was around ten times lower in the groundwater fraction. These observations were reinforced by the results of the purging experiment conducted at both field sites, which showed that generally less DNA was recovered after extensive purging (e.g. 6 well volumes) and in many cases the *ethB* gene copy number was higher in the early purges (1–3 well volumes) compared with 6 well volumes. This reflects the progressive removal of the sediment fraction from the groundwater sample with continued purging. It suggests that extended purging of monitoring wells prior to groundwater sampling, as supported within current site sampling protocols (e.g. Nielsen and Nielsen 2006; USEPA 2017), should be avoided if the aquifer ETBE biodegradation potential is assessed by the detection of the *ethB* gene. However, obtaining true aquifer samples is essential to avoid capturing samples that provide misleading results, i.e. well storage samples (Bonte et al. 2017). Hence, a balance between purging and aquifer sediment recovery must be achieved. Therefore, it is recommended that purging should be conducted until field parameters stabilise prior to sample collection. In the current study, less than 3 purge volumes were removed prior to sampling. Sampling regimes should emphasise the collection of aquifer sediment to prioritise the analysis of the attached microbial community for the presence of the *ethB* gene, according to the options outlined in the “Groundwater monitoring to evaluate ETBE-biodegradation” section. Considering practicality and cost, a mixed groundwater-sediment slurry sample obtained from monitoring wells after minimal purging can provide sufficient aquifer sediment for microbiological analysis of ETBE biodegradation potential via *ethB* gene detection.

Interestingly, ETBE was not detected in the last groundwater survey of both monitoring wells at Site F (December 2016), but was detected previously at 1.4 mg L^-1^ in well F2 (October 2016). Given that the field samples used in this study were collected in February 2019, the absence of ETBE at that time further supports the conclusion that the ETBE-degraders are attached to the aquifer sediment. This is because planktonic organisms would be redistributed by the groundwater flow, whereas the *ethB* gene-containing organisms would remain attached to the aquifer matrix at the location of this monitoring well. Furthermore, given that ETBE was not detected in groundwater for several years, this suggests that ETBE-degrading organisms persist in the aquifer once established, as reported by Nicholls et al. (2020).

### Geographical distribution of ETBE-degrading organisms at gasoline-release sites

The organisms identified as ‘responders’ from the microcosm study in Nicholls et al. (2020) were compared with the sequencing data obtained from the Site F field samples in this study. In Nicholls et al. (2020) the microcosms were originally established using aquifer sediment and groundwater from Site F, and the same monitoring wells (F1 and F2) were re-sampled in the present study. The microcosm study identified OTUs predominantly belonging to the Comamonadaceae that increased in relative abundance when exposed to ETBE. The authors therefore hypothesised that the ‘responders’ were involved in aerobic biodegradation of ETBE. The re-sampling of Site F revealed that ~4 % of the microbial community from well F2 were the same ‘responders’ that were identified from the microcosm study (Supplementary Figure 4), with most belonging to the Comamonadaceae. Interestingly, a study investigating the bacterial colonisation of pristine sediments reported that members of the Comamonadaceae were early colonisers and therefore dominated the final microbial communities (Fillinger et al. 2019). Furthermore, in a recent study, van der Waals et al. (2019) identified Comamonadaceae as an abundant bacterial group in a mixed algal-bacterial culture obtained from contaminated groundwater that biodegraded ETBE in a batch reactor, suggesting that these microorganisms may play an important role in ETBE metabolism. To assess if the same microcosm ‘responders’ were identifiable at another ETBE-release site, this data was compared with Site T field data. At Site T the *ethB* gene was detected in well T1 but low numbers of ETBE-responders were evident and, conversely, where no *ethB* gene was detected ETBE-responders were most dominant, with these organisms primarily belonging to the Gammaproteobacteria (well T3). This result suggests that the detection of specific organisms is less important that the detection of the degradative gene (*ethB*), i.e. that taxonomic analysis of a given microbial community is less reliable than the analysis of functional capability, based on the detection of the *ethB* gene within the consortia. Hence, the taxonomic composition of the microbial community may not indicate ETBE biodegradation potential. This has been attributed to the functional redundancy of ETBE-degrading consortia, in that the distribution of the *ethB* gene within the microbial community may be independent of its taxonomic composition (Kyselková et al. 2019). It implies that many bacterial species in a consortium could facilitate ETBE biodegradation, according to their functional capability. The exchange of genetic traits in a biofilm environment is considered advantageous in conserving degradative function (Singh et al. 2006; Rizoulis et al. 2013). In the case of ETBE the *eth* operon is flanked by two identical transposons and is readily lost under non-selective conditions (Chauvaux et al. 2001). Therefore, close proximity of microbes in a biofilm could facilitate the horizontal transfer of the *eth* gene cluster between different bacterial species within the attached microbial community (Singh et al. 2006; Kyselková et al. 2019).

## Conclusion

This study has shown that under both laboratory and field conditions ETBE-degrading organisms in aquifers may preferentially attach to the aquifer substratum. This result has important implications for (i) sampling protocols aimed at characterising the aquifer ETBE biodegradation potential at ETBE-release sites, and (ii) the performance assessment of management measures such as natural attenuation or bioremediation, using microbiological-based analyses of ETBE biodegradation. In this context, the analysis of the *ethB* gene in the aquifer microbial community can provide additional evidence to support the hydrochemical assessment of aerobic ETBE biodegradation in groundwater at ETBE-release sites. The detection of the *ethB* gene in samples is a better function-based indicator of the aquifer ETBE biodegradation potential than the taxonomic composition of the microbial community. However, the evidence from this study indicates strongly that sampling solely the planktonic community in groundwater is not a reliable measure of the presence of ETBE-degrading organisms, nor an indicator of ETBE biodegradation potential in the aquifer microbial community. The low DNA recovery from the planktonic phase found in this study suggests large volumes (e.g. >2 L) of groundwater would be required for *ethB* gene detection, where no aquifer sediment is available. Furthermore, even when the *ethB* gene is detected in groundwater, its relative abundance is approximately ten-fold less than in the attached community, which may result in misleading inferences on ETBE biodegradation potential. Therefore, sampling of the attached microbial community should be prioritised for this microbiological assessment. A groundwater-aquifer sediment slurry sample obtained from a pumped monitoring well can provide a sample of the aquifer microbial community for the analysis of ETBE biodegradation potential and temporal development of the ETBE-degrading community. This sample should be collected after minimal purging of the monitoring well once field parameters have stabilised, to ensure a representative well sample is obtained and to maximise the recovery of the aquifer sediment fraction, and therefore DNA yield and detection of the *ethB* gene. This is a relatively rapid and cost-effective method to obtain samples for this microbiological assessment.

## Supplementary information

Table S1 (DOCX 14 kb)

Fig. S1 (DOCX 1883 kb)

Fig. S2 (DOCX 93 kb)

Fig. S3 (DOCX 221 kb)

Fig. S4 (DOCX 222 kb)

## Data Availability

The datasets generated and analysed during the current study are available in the SRA repository, PRJNA720508, which can be accessed via https://www.ncbi.nlm.nih.gov/sra/PRJNA720508.
