# Supplementary material for: Distribution of ETBE-degrading microorganisms and functional capability in groundwater, and implications for characterising aquifer ETBE biodegradation potential
Source: Environ Sci Pollut Res Int. 2021 Aug 4;29(1):1223–38. doi: 10.1007/s11356-021-15606-7 (PMC8724112; doi:10.1007/s11356-021-15606-7)
Supplement: Supplementary file 1 — (DOCX 14 kb) [file 11356_2021_15606_MOESM1_ESM.docx]

| Monitoring well | F1 | | F2 | | | T1 | | | T2 | | | T3 | | |
| --- | --- | --- | --- | --- | --- | --- | --- | --- | --- | --- | --- | --- | --- | --- |
| Purge Volume | 1 | 3 | 1 | 3 | 6 | 1 | 3 | 6 | 1 | 3 | 6 | 1 | 3 | 6 |
| F (mg L^-1^) | <0.01 | <0.01 | <0.01 | <0.01 | <0.01 | <0.01 | <0.01 | <0.01 | <0.01 | <0.01 | <0.01 | <0.01 | <0.01 | <0.01 |
| Cl (mg L^-1^) | 32.77 | 33.74 | 29.57 | 28.81 | 29.42 | 369.6 | 365.5 | 371.6 | 161.3 | 161.8 | 165.8 | 290.4 | 271.6 | 268.1 |
| SO_4_ (mg L^-1^) | 45.50 | 45.56 | 38.36 | 36.43 | 38.70 | 54.45 | 27.24 | 21.56 | 41.48 | 40.01 | 39.17 | 119.09 | 124.2 | 126.7 |
| NO_3_ (mg L^-1^) | 42.86 | 34.88 | 24.36 | 22.27 | 39.80 | 2.38 | 2.66 | 2.33 | 19.02 | 56.97 | 18.37 | 2.96 | 2.34 | 2.69 |
| PO_4_ (mg L^-1^) | <0.30 | <0.30 | <0.30 | <0.30 | <0.30 | <0.30 | <0.30 | <0.30 | <0.30 | <0.30 | <0.30 | <0.30 | <0.30 | <0.30 |
| NH_4_ (mg L^-1^) | <0.10 | <0.10 | <0.10 | <0.10 | <0.10 | 3.81 | <0.10 | <0.10 | <0.10 | <0.10 | <0.10 | <0.10 | <0.10 | <0.10 |
| K (mg L^-1^) | 2.72 | 2.76 | 2.29 | 2.16 | 2.27 | 11.20 | 11.50 | 11.90 | 8.82 | 8.83 | 8.83 | 14.11 | 13.22 | 13.18 |
| Mg (mg L^-1^) | 19.60 | 18.62 | 19.23 | 18.51 | 19.15 | 54.77 | 54.27 | 56.49 | 17.76 | 18.87 | 19.29 | 50.52 | 46.38 | 45.68 |
| Ca (mg L^-1^) | 122.23 | 48.86 | 101.87 | 103.84 | 117.57 | 85.81 | 69.33 | 54.81 | 44.56 | 45.19 | 45.76 | 67.94 | 66.28 | 64.27 |

Table S1. Analysis of dissolved inorganic compounds measured in groundwater sampled after removal of different purge volumes from monitoring wells at Site F and Site T.
