# Supplementary material for: Distribution of ETBE-degrading microorganisms and functional capability in groundwater, and implications for characterising aquifer ETBE biodegradation potential
Source: Environ Sci Pollut Res Int. 2021 Aug 4;29(1):1223–38. doi: 10.1007/s11356-021-15606-7 (PMC8724112; doi:10.1007/s11356-021-15606-7)
Supplement: Supplementary file 2 — (DOCX 1883 kb) [file 11356_2021_15606_MOESM2_ESM.docx]

**
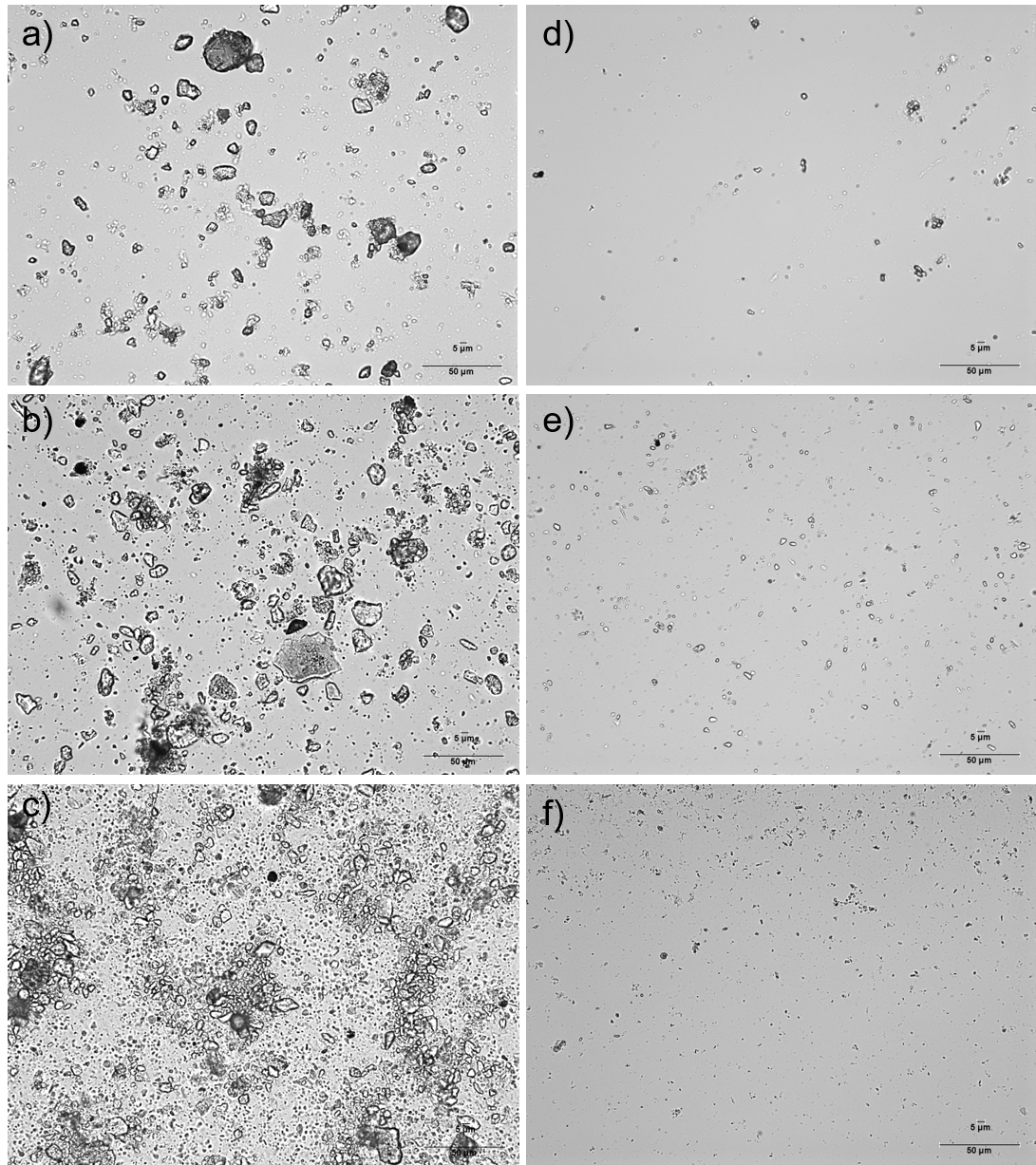
**

Figure S1. Sediment grain images for the 5 µm filter confirming this membrane captured large sediment grains (a-c), although some small sediment particles (<5 µm) passed through and were captured on the 0.2 µm membrane (d-f). A 50 µm and 5 µm scale bar is included for size reference.
