# Supplementary material for: Distribution of ETBE-degrading microorganisms and functional capability in groundwater, and implications for characterising aquifer ETBE biodegradation potential
Source: Environ Sci Pollut Res Int. 2021 Aug 4;29(1):1223–38. doi: 10.1007/s11356-021-15606-7 (PMC8724112; doi:10.1007/s11356-021-15606-7)
Supplement: Supplementary file 3 — (DOCX 93 kb) [file 11356_2021_15606_MOESM3_ESM.docx]

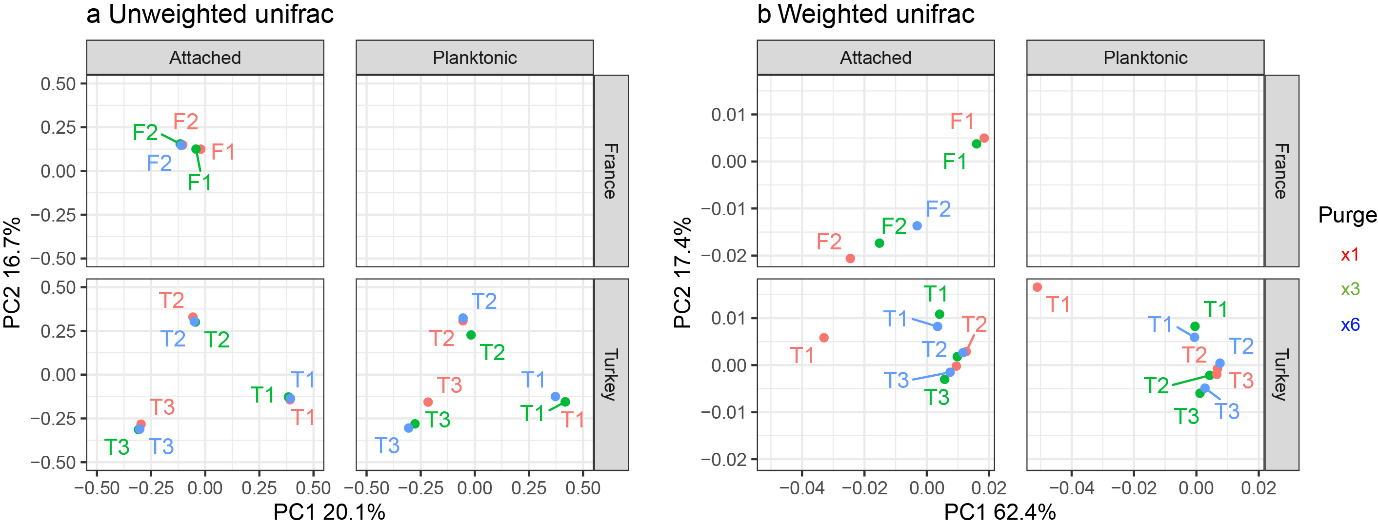


Figure S2. Principal components analysis using a) unweighted and b) weighted Unifrac distances of purged samples (attached and planktonic) from Site F and Site T. The attached and planktonic samples for each site have been separated for clarity but are shown on the same scale. Colour represents purge volume.
