# Supplementary material for: Distribution of ETBE-degrading microorganisms and functional capability in groundwater, and implications for characterising aquifer ETBE biodegradation potential
Source: Environ Sci Pollut Res Int. 2021 Aug 4;29(1):1223–38. doi: 10.1007/s11356-021-15606-7 (PMC8724112; doi:10.1007/s11356-021-15606-7)
Supplement: Supplementary file 4 — (DOCX 221 kb) [file 11356_2021_15606_MOESM4_ESM.docx]

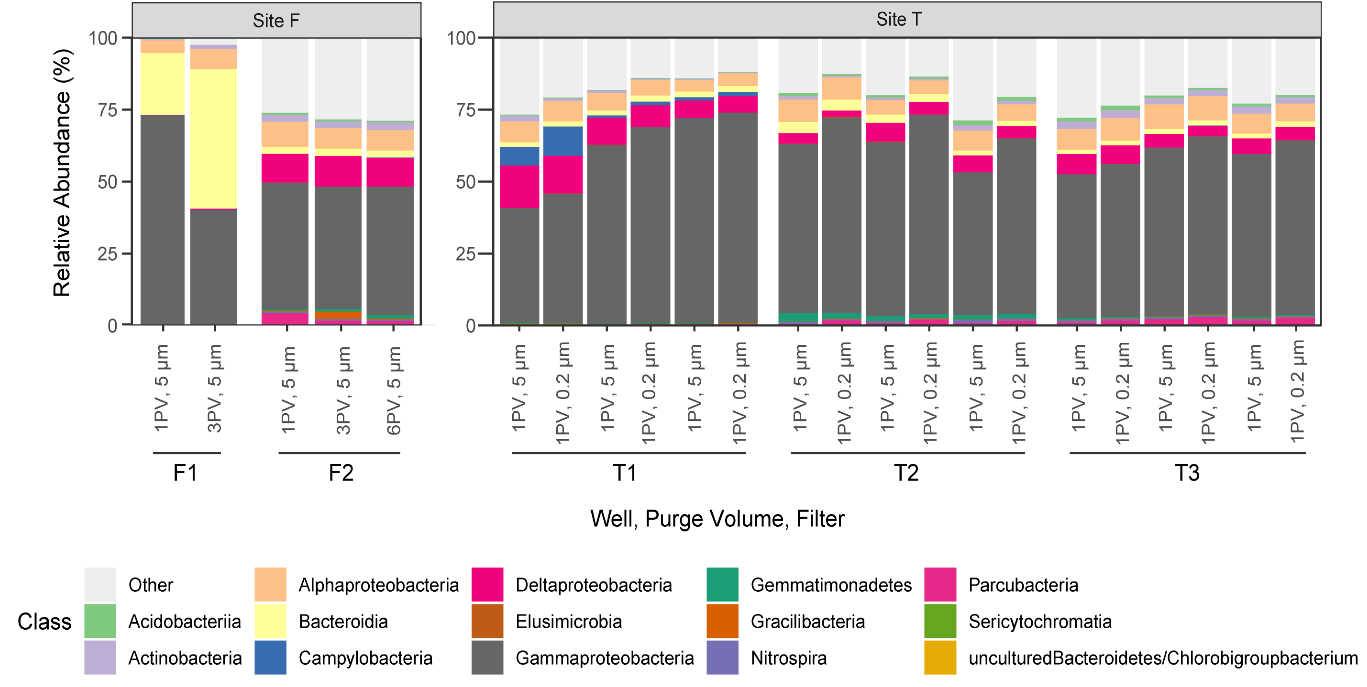


Figure S3. Relative abundance of OTUs at the class level. Classes with a relative abundance of less than 1 % have been categorised as ‘Other’ for clarity. Note: Taxonomic assignments were made against the Silva 132 database (see Methods and section 3.2).
