# Supplementary material for: Distribution of ETBE-degrading microorganisms and functional capability in groundwater, and implications for characterising aquifer ETBE biodegradation potential
Source: Environ Sci Pollut Res Int. 2021 Aug 4;29(1):1223–38. doi: 10.1007/s11356-021-15606-7 (PMC8724112; doi:10.1007/s11356-021-15606-7)
Supplement: Supplementary file 5 — (DOCX 222 kb) [file 11356_2021_15606_MOESM5_ESM.docx]

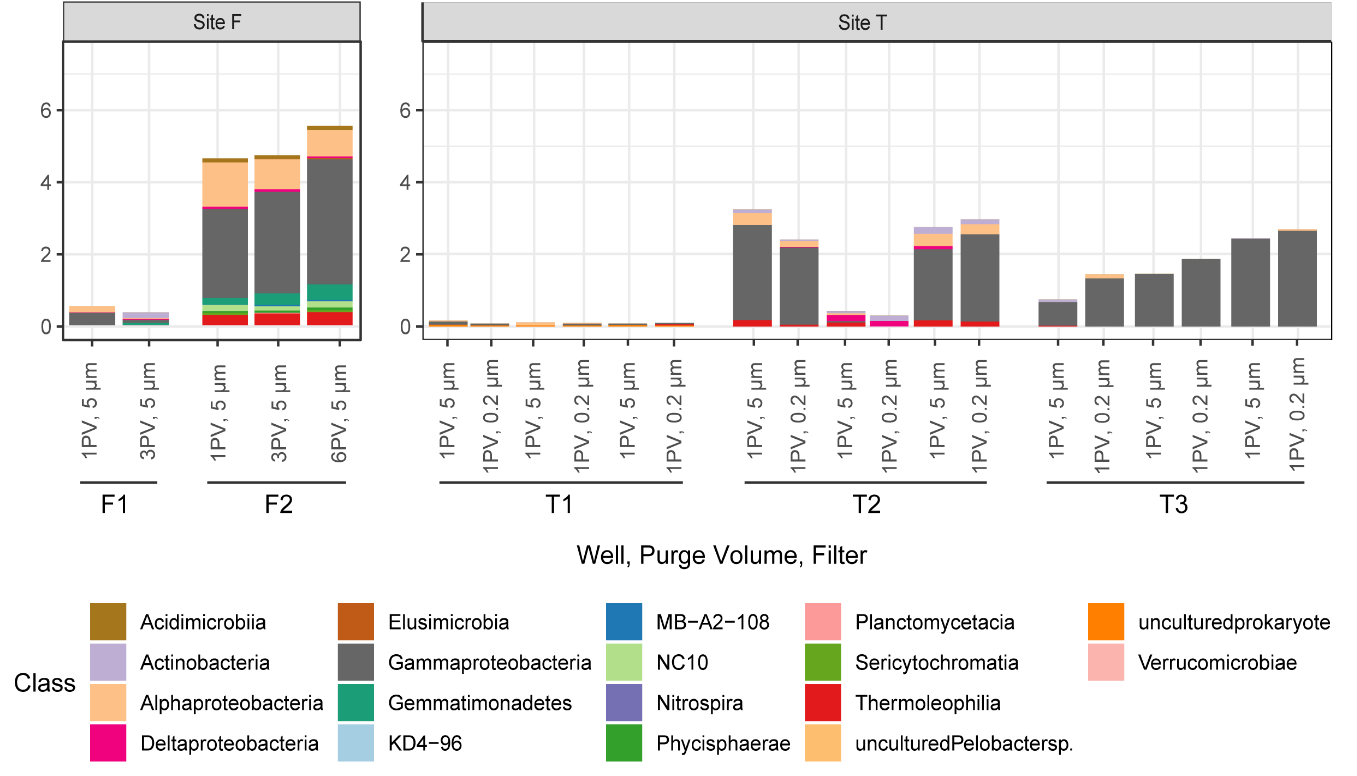


Figure S4. Relative abundance of OTUs at the class level for Site F and Site T that were identified as responders to ETBE additions in the microcosm study from Nicholls et al. (2020). Note: Taxonomic assignments were made against the Silva 132 database (see Methods and section 3.2).
